# Mismatch Negativity and P300 in Children With Attention-Deficit/Hyperactivity Disorder (ADHD): A Comparative Event-Related Potential Study Using Healthy Siblings as Controls

**DOI:** 10.7759/cureus.93078

**Published:** 2025-09-23

**Authors:** Rohit Saroha, Muneeb Kosvi, Soni Singh

**Affiliations:** 1 Physiology, Santosh Deemed to be University, Ghaziabad, IND; 2 Physiology, Teerthanker Mahaveer University, Moradabad, IND

**Keywords:** adhd, erps, executive functioning impairments, mmn, neurophysiological markers, p300

## Abstract

Aim and background: Attention-deficit/hyperactivity disorder (ADHD) is a neurodevelopmental disorder that involves attention, impulse control, and/or executive function difficulties. Knowledge of the neural bases underlying ADHD is essential for developing accurate diagnostic and treatment methods. This study assessed cognitive dysfunction in children with ADHD with respect to endophenotypic traits of event-related potentials (ERPs), specifically the mismatch negativity (MMN) and P300 components, and compared them to those of healthy siblings. These ERP components serve as neurophysiological markers of pre-attentive and attentive cognitive processes, respectively.

Materials and methods: This observational cross-sectional study was conducted at Lady Hardinge Medical College, New Delhi, India, from November 2016 to January 2021. The study group comprised 18 children diagnosed with ADHD, who were compared to 10 healthy siblings serving as controls. ERPs were recorded using the SCHWARZER TOPAS EMG neurophysiological system (Schwarzer GmbH, Munich, Germany), which was triggered via a PC running the stimulus delivery software during an oddball paradigm of auditory stimuli. Post hoc pairwise comparisons across different electrode locations were used to evaluate the MMN and P300 components relative to their delays and amplitudes. Independent t-tests were used to assess differences in MMN and P300 latency and amplitude between the groups, with Cohen's d calculated to assess effect size.

Results: Children with ADHD showed significantly prolonged latencies and reduced amplitudes in both the MMN and P300 components compared to their healthy controls. For example, MMN latency at Fz was 231.83±12.69 ms in the ADHD group and 196.60±11.53 ms in controls (P < 0.05), showing a strong effect size of 2.66, indicating a substantial difference between the two groups. In addition, there was a significant difference in the amplitude of P300 at the Fz site (µV) between ADHD children (4.28 ± 1.84) and controls (11.20 ± 3.29; p < 0.0001), with a large effect size of 2.98, favoring the control group. There were no correlations between Connors' ADHD Rating Scale subscores and any of the ERP variables.

Conclusion: These results indicate that children with ADHD demonstrate significant neurophysiological differences in MMN and P300 components compared to their healthy siblings. While ERPs show potential as objective and quantifiable markers of cognitive dysfunction in ADHD, their utility as standalone diagnostic indices remains limited, especially given this study's small sample size and absence of correlation with behavioral severity. Larger studies are needed to confirm and extend these findings before ERPs can be reliably used in clinical diagnostics for ADHD.

## Introduction

Attention-deficit/hyperactivity disorder (ADHD) is commonly diagnosed in children and often persists into adulthood. It is associated with a combination of persistent and pervasive patterns that deviate from the normal experiences of children during their developmental stages, such as inattention, overactivity, or impulsivity. It is estimated that ADHD affects 5-10% of children worldwide (prevalence varies according to diagnostic criteria and the community studied) [1,2]. The disorder poses formidable and critical challenges not only to children diagnosed with ADHD but also to their families, educators, and the healthcare system, resulting in considerable genetic, neurological, and environmental variables [3].

Recent meta-analyses have revealed that ADHD is characterized by distinct structural and functional changes in the prefrontal cortex, frontoparietal and limbic networks, and the corpus callosum, with delayed neurodevelopmental trajectories particularly evident in children [4-6]. Modern neuroimaging shows alterations in functional connectivity, especially disruptions between the DMN and TPN, which contribute to deficits in attention and executive function [7]. Advances in electrophysiological methods, biochemical markers, and artificial intelligence-driven diagnostics are rapidly enhancing the objectivity and personalization of ADHD assessment [8,9]. Nonetheless, the disorder's neurobiological basis remains heterogeneous, with a continued need for larger, well-designed studies [7].

One strategy to improve our understanding of neurobiological-cognitive markers for ADHD is the use of event-related potentials (ERPs), which are the time-locked components elicited by specific sensory, cognitive, or motor events that are measured with electroencephalography (EEG) [10]. ERPs offer a non-invasive method for studying the electrical signals of brain activity with temporal resolution and have been broadly used to study various cognitive processes [11]. The mismatch negativity (MMN) and P300 are particularly helpful for ADHD experiments in different laboratories [12]. MMN involves pre-attentive processing and helps in measuring the brain's inherent reaction to changes in auditory input, whereas P300 resolves with attentional signals and the distribution of cognitive resources towards pertinent stimuli/tasks [13].

The MMN component, usually elicited in an auditory oddball paradigm, is a fronto-central negativity that occurs approximately 100-250 ms after stimulus onset [14]. It should also act as an immediate separator between the acoustic input and sensory memory for prior stimuli presented [15]. Previous studies on ADHD have demonstrated that MMN amplitudes are typically lower, while latencies are longer; thus, this index indicates pre-attentive dysfunction or defective auditory differentiation ability [16]. These results suggest that children with ADHD may have a reduced capacity to automatically discriminate shifts in their auditory surroundings, which may be related to the attention experienced by children with the disorder [10].

Similarly, the P300 component elicits deviant and noticeable stimuli during an oddball task and usually has a latency of 300-600 ms [11]. The P300 amplitude is thought to index attentional resource allocation and the updating process in working memory, whereas its latency supposedly reflects a speed estimate for cognitive processing [13]. Reduced P300 amplitude and prolonged latency in children with ADHD provide evidence of attentional control deficits, such as impairments in working memory and cognitive processing speed [15]. These ERP abnormalities have been suggested as neural correlates of behavioral issues characteristic of ADHD, including distractibility, impulsivity, and inefficient attention control [12].

Differences in ERPs observed across studies may be attributed to alterations in study design, participant demographics, and varying experimental paradigms enacted to elicit ERP responses [16]. Another limitation is that ERP components can be affected by factors such as age, cognitive load, and concurrent disorders, which might affect the amplitude or latency of ERP responses detected [1]. Therefore, it is necessary to standardize the ERP approach and ensure that the results are reproducible across different populations and conditions [4].

Related studies

According to Yamamuro et al., ERPs, particularly the P300 and MMN components, are dissociable central physiological abnormalities that are implicated in ADHD cognitive dysfunction. Their results, involving 51 children with ADHD and 15 controls, suggested that children with ADHD had significantly delayed P300 latencies, with smaller amplitudes contrasted with having regulations. Decreased MMN component amplitude in children with ADHD, especially at central electrodes all over the scalp (e.g., Pz), suggests reduced pre-attentive information processing even when their concentration remained unchanged compared to controls and might support behavioral observations of continuous distraction. This attenuation correlates with the severity of ADHD symptoms, suggesting that MMN may serve as a useful biomarker for assessing the level of ADHD impairment. They concluded that ERPs, particularly P300 and N2b, grouped within the MMN component, were capable of offering new insights into cognitive alterations associated with ADHD spectrum disorders and can be used as diagnostic adjuncts for the comprehensive monitoring of this disorder [17].

Kemner et al. examined cognitive impairment underlying ERPs in ADHD. Even when task relevance was very high, 20 children with ADHD and matched controls of the same number showed striking reductions in P300 amplitudes across a variety of circumstances. These results suggest that ADHD may manifest as a general deficit in cognitive processing as opposed to deficits specific to individual tasks. The study also highlighted that attenuated P300 amplitudes were evident in children with ADHD, even when tasks did not require active attention, supporting the notion that these ERP deficits represent a central feature of the neurobiology of the disorder. This study underscores the importance of employing ERPs to understand cognitive deficits in ADHD [18].

While Holcomb et al. (1985) [19] provided early insights into ERP development in ADHD, recent longitudinal studies have expanded these findings. For instance, Münger et al. (2022, 2023) observed that children with ADHD exhibit persistent P300 alterations over several years, with prolonged latencies and decreased amplitudes indicating delayed maturation of attentional circuits [20,21]. Similarly, Doehnert et al. (2013) reported divergent neurodevelopmental ERP trajectories, emphasizing that deficits in P300 components may evolve across adolescence, supporting the notion that ADHD-related cognitive impairments progress with age and involve delays in developmentally sensitive brain circuits [22].

ADHD is a complex neuropsychiatric condition that has a profound impact on cognitive and behavioral performance. ERPs, particularly MMN and P300, may provide useful information regarding the neural underpinnings of ADHD as objective indices of cognitive processing deficits. In the present study, we aimed to determine the electrophysiological characteristics of cognitive impairments in ADHD and to compare ERP components between children with ADHD and healthy siblings. This will help increase the growing amount of research on ERPs as biomarkers for ADHD, ultimately contributing to improved diagnostic and treatment strategies.

## Materials and methods

Study design

This observational cross-sectional study was conducted at the Department of Physiology, in collaboration with the Department of Psychiatry, Lady Hardinge Medical College, from November 2016 to January 2021. The purpose of this research was to study differences in cognitive processing between children with ADHD and their healthy siblings using ERPs.

Study population

Of the initial 37 children recruited, nine were excluded due to excessive movement artifacts, a necessary step to ensure data quality, but one that may have led to a sample biased toward children with less severe hyperactivity symptoms. Consequently, the final cohort consisted of 18 children with ADHD and 10 healthy siblings, with demographic details such as gender, socioeconomic status, and educational background recorded. These variables can influence ERP outcomes and symptom variability, highlighting the heterogeneous and multifactorial nature of ADHD, which involves complex interactions between biological, psychological, and social factors. The final sample size of 28 participants was limited by these exclusion criteria and the disruptions caused by the COVID-19 pandemic. Despite its modest size, the study observed substantial effect sizes, suggesting sufficient power to detect meaningful group differences. Given the exploratory and observational design, a formal a priori power calculation was not feasible, but future research with larger, more representative samples and planned power analyses is warranted to validate and extend these findings.

Methods

This observational cross-sectional study included children aged 6-12 years who visited the outpatient department or who were admitted to the wards of Lady Hardinge Medical College.

Inclusion and exclusion criteria

Children were identified as having ADHD based on the DSM-5 (Diagnostic and Statistical Manual of Mental Disorders, 5th Edition) criteria [23]. Newly diagnosed patients aged 6-12 years were included in this study. A single control group was established, consisting of siblings of children with ADHD matched for age. A screening procedure to exclude individuals with subclinical ADHD traits was conducted to control for potential confounding factors related to ADHD symptoms in the control group. The exclusion criteria were concomitant psychiatric disorders, severe visual or auditory impairments, systemic diseases, and recent medication use.

ERP recording

ERPs were recorded using the SCHWARZER TOPAS EMG system during an auditory oddball paradigm, as recommended in ERP research standards [24]. Electrodes were placed at Fz, Cz, C3, and C4 with a reference electrode on the mastoid. MMN was evoked by infrequent auditory stimuli, and the P300 component was used for infrequent targets. The data were averaged (combining epochs for intervals during which the participant performed only two consecutive correct responses) after artifact rejection. Latencies and amplitudes of the MMN and P300 components were calculated.

Statistical analysis

Data analysis was performed using IBM SPSS Statistics for Windows, Version 26 (Released 2019; IBM Corp., Armonk, New York). Continuous variables are expressed as mean ± SD and were compared using an unpaired t-test. Spearman's correlation coefficient was calculated to explore the relationship between the connection scale and ERP components. Statistical significance was defined as p<0.05. Given multiple comparisons across electrode sites and ERP parameters, the risk of false positives is acknowledged. Although formal correction methods for multiple testing (e.g., the Bonferroni or False Discovery Rate (FDR) methods) were not applied due to the exploratory nature and sample size constraints, findings should be interpreted with caution. Future studies with larger samples should apply appropriate corrections to control for Type I errors.

## Results

**Figure 1:**
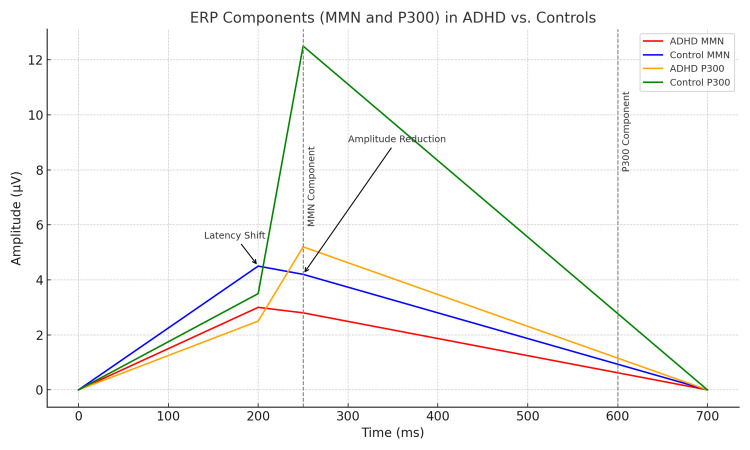
ERP components (MMN and P300) in children with ADHD versus controls The MMN and P300 components are plotted with respect to time (in milliseconds) and amplitude (in microvolts). Vertical dashed lines represent typical latency markers for MMN (~250 ms) and P300 (~600 ms). ERP: event-related potential; MMN: mismatch negativity; ADHD: attention-deficit/hyperactivity disorder

This observational cross-sectional study found marked differences in ERPs (Figure 1) between children with ADHD and their neurotypical siblings. Of the 37 children initially recruited, nine were excluded due to excessive movement artifacts, resulting in a final sample of 18 ADHD subjects and 10 controls. This study employed auditory ERPs to evaluate cognitive function with a particular focus on the MMN and P300 components.

MMN findings

Compared to controls, MMN latency was much slower in patients with ADHD. For example, the mean delay on the FZ site was 231.83 ± 12.69 ms in the ADHD group versus 196.60 ± 11.53 ms in controls (p < 0.0001). Concomitantly, the MMN latency at the Cz, C3, and C4 sites was also significantly prolonged in the AD/HD participants (p < 0.0001). With respect to amplitude, children with ADHD showed significantly smaller MMN amplitudes at all electrodes examined in the analysis, e.g., at the Fz site, the mean amplitude was 3.17 ± 1.79 µV at Fz vs. 5.10 ± 1.29 µV in controls (p = 0.0059). The results were comparable for Cz, C3, and C4 (p<0.0008, p<0.0001, and p=0.0013, respectively).

P300 results

The P300 component is another sign of significant alterations in children with ADHD compared with controls. In children with ADHD, there was a markedly prolonged latency at all examined sites of the P300 wave. In the example shown in the study, when we compared the Fz location of the P300, the delay was significantly longer in the ADHD group (375.44 ± 27.20 ms) compared to the control group (p<0.0001; 317.40 ± 19.62 ms). In children with ADHD, the amplitude of P300 was significantly reduced to 4.28 ± 1.84 µV at Fz compared to control subjects who showed an amplitude of 11.20 ± 3.29 µV (p<0.0001). Thus, these findings demonstrate that there is a significant relationship between cognitive processing and improper distribution of brain resources among children with ADHD.

Correlation analysis

There were no significant correlations between MMN and P300 latency or amplitude at any electrode location with either connection. The absence of a link between ERPs and ADHD symptom severity, as reported by parents, suggests that the abnormal neurophysiological processes revealed by ERPs may not directly reflect the symptom severity rated by parents.

Tables 1, 2 list the MMN latency and amplitude for the ADHD and control groups, respectively. Accounting for the MMN latency between the different electrode locations, it was evident that the ADHD group had a considerably delayed latency, with p-values less than 0.0001 for all comparisons. Table 2 shows the lower MMN amplitude in the ADHD group compared to the control group, with test statistic values ranging from 0.001 to the extent that participants had a mental age of less than eight years (P < 0.0001). To support this statement, Tables 3, 4 provide a detailed assessment of the P300 component, indicating that the ADHD group had a significantly larger P300 latency duration and a smaller P300 amplitude than the control group. All p-values for these findings were less than 0.0001, indicating strong statistical significance.

**Table 1:** MMN latency (ms) comparison A p-value <0.05 is considered statistically significant. ADHD: attention-deficit/hyperactivity disorder; MMN: mismatch negativity

Parameter	ADHD Group (Mean ± SD)	Control Group (Mean ± SD)	p-value
MMN Fz latency	231.83 ± 12.69	196.60 ± 11.53	<0.0001
MMN Cz latency	224.06 ± 11.91	199.70 ± 17.67	0.0002
MMN C3 latency	226.06 ± 12.07	195.20 ± 8.12	<0.0001
MMN C4 latency	226.56 ± 11.01	198.10 ± 6.98	<0.0001

**Table 2:** MMN amplitude (µV) comparison A p-value <0.05 is considered statistically significant. ADHD: attention-deficit/hyperactivity disorder; MMN: mismatch negativity

Parameter	ADHD Group (Mean ± SD)	Control Group (Mean ± SD)	p-value
MMN Fz amplitude	3.17 ± 1.79	5.10 ± 1.29	0.0059
MMN Cz amplitude	2.50 ± 1.47	5.00 ± 2.00	0.0008
MMN C3 amplitude	2.17 ± 1.42	5.10 ± 1.37	<0.0001
MMN C4 amplitude	2.39 ± 1.46	4.70 ± 1.89	0.0013

**Table 3:** P300 latency (ms) comparison A p-value <0.05 is considered statistically significant. ADHD: attention-deficit/hyperactivity disorder; MMN: mismatch negativity

Parameter	ADHD Group (Mean ± SD)	Control Group (Mean ± SD)	p-value
P300 Fz latency	375.44 ± 27.20	317.40 ± 19.62	<0.0001
P300 Cz latency	383.28 ± 21.45	312.80 ± 16.69	<0.0001
P300 Pz latency	374.44 ± 29.40	308.90 ± 18.39	<0.0001

**Table 4:** P300 amplitude (µV) comparison A p-value <0.05 is considered statistically significant. ADHD: attention-deficit/hyperactivity disorder; MMN: mismatch negativity

Parameter	ADHD Group (Mean ± SD)	Control Group (Mean ± SD)	p-value
P300 Fz amplitude	4.28 ± 1.84	11.20 ± 3.29	<0.0001
P300 Cz amplitude	5.28 ± 2.35	13.20 ± 2.49	<0.0001
P300 Pz amplitude	4.78 ± 2.16	12.80 ± 1.48	<0.0001

This study highlights the significant neurophysiological deficits in children with ADHD that manifest as delayed MMN and P300 latencies, accompanied by reduced amplitudes in these responses, which are thought to be manifestations of cognitive problems. These findings highlight the potential of ERPs as objective indicators of cognitive dysfunction in patients with ADHD. However, the lack of an association with severity suggests that further research is needed to investigate this relationship [25].

The t-test results (Table 5) confirmed that the differences in MMN and P300 latencies and amplitudes between children with ADHD and controls were statistically significant across all electrode sites. The effect size calculations showed that the differences were large across all comparative groups, highlighting their magnitude (Table 6).

**Table 5:** Independent t-test analysis A p-value <0.05 is considered statistically significant. ADHD: attention-deficit/hyperactivity disorder; ERP: event-related potential; MMN: mismatch negativity

ERP Component	Electrode Site	Parameter	t-value	p-value	Significant Difference
MMN	Fz	Latency (ms)	7.36	<0.0001	Yes
		Amplitude (µV)	-3.06	0.0059	Yes
	Cz	Latency (ms)	5.51	<0.0001	Yes
		Amplitude (µV)	-4.03	0.0008	Yes
	C3	Latency (ms)	7.70	<0.0001	Yes
		Amplitude (µV)	-5.35	<0.0001	Yes
	C4	Latency (ms)	7.96	<0.0001	Yes
		Amplitude (µV)	-3.97	0.0013	Yes
P300	Fz	Latency (ms)	6.39	<0.0001	Yes
		Amplitude (µV)	-7.72	<0.0001	Yes
	Cz	Latency (ms)	8.10	<0.0001	Yes
		Amplitude (µV)	-8.19	<0.0001	Yes
	Pz	Latency (ms)	6.58	<0.0001	Yes
		Amplitude (µV)	-12.06	<0.0001	Yes

**Table 6:** Effect size calculation (Cohen's d) ERP: event-related potential; MMN: mismatch negativity

ERP Component	Electrode Site	Parameter	Cohen's d	Effect Size
MMN	Fz	Latency (ms)	2.66	Large
		Amplitude (µV)	1.55	Large
	Cz	Latency (ms)	2.30	Large
		Amplitude (µV)	1.93	Large
	C3	Latency (ms)	2.78	Large
		Amplitude (µV)	2.38	Large
	C4	Latency (ms)	2.87	Large
		Amplitude (µV)	1.75	Large
P300	Fz	Latency (ms)	2.41	Large
		Amplitude (µV)	2.98	Large
	Cz	Latency (ms)	2.91	Large
		Amplitude (µV)	3.18	Large
	Pz	Latency (ms)	2.46	Large
		Amplitude (µV)	4.56	Large

## Discussion

The researchers discovered significant neurophysiological anomalies in ERPs between children with ADHD and their healthy siblings, which are indicative of ADHD-related cognitive deficits. As observed in the present study, prolonged MMN and P300 latencies with attenuated amplitudes suggest impairment in both pre-attentive (MMN) and attentive (the parietal-generated 'late' positivity, centered over CPz) processing pathways that may be characteristic of ADHD, although this requires confirmation in larger, independently replicated studies [26]. In line with the present study, these findings suggest that ADHD is defined by anomalies in processing information and regulating attention [27]. The lack of a significant association between the Connors' ADHD Rating Scale scores and ERP parameters reflects a complicated ADHD pathology and openly demonstrates that ERPs may reflect biomarkers for specific aspects or endophenotypes of cognitive deficits of cognitive dysfunction rather than direct behavioral symptom severity [25].

Comparative analysis

In comparison to the present results with earlier studies, in line with previous research, our findings support the idea that there are abnormalities in ERP to everyday life stimuli; indicative alterations at basic sensory levels can be detectable on a clinical basis. A study by Yamamuro et al. [17] found delayed latencies of the P300 and amplitude reductions in children with ADHD, similar to our findings. They also discovered that MMN amplitudes in these children were reduced, particularly at the central electrode site (Pz), which corresponds to our finding of a decreased magnitude of MMN amplitudes across multiple sites during response-accuracy control. The analysis conducted by Kemner et al. [18] additionally provides supporting evidence for this, with results showing that reductions in P3 amplitudes in children with ADHD occurred reliably regardless of task relevance. These observations further corroborate the interpretation that the ERP abnormalities primarily reflect altered processing of stimulus deviance, rather than general differences between the groups. Additionally, recent longitudinal ERP research by Münger et al. (2022, 2023) and Doehnert et al. (2013) documented prolonged P3 latencies and diminished amplitudes in ADHD, aligning with our current findings of delayed ERP components and reinforcing the notion of persistent neurodevelopmental alterations in attentional processing in ADHD [20-22]. A detailed comparative analysis is presented in Table 7.

**Table 7:** Comparative analysis ADHD: attention-deficit/hyperactivity disorder; MMN: mismatch negativity

Study	Key Findings	Similarity With Current Study
Yamamuro et al. [17]	Prolonged P300 latency, reduced amplitude; attenuated MMN	Similar prolonged latencies and reduced amplitudes in both MMN and P300
Kemner et al. [18]	Smaller P3 amplitudes regardless of task relevance	Similar findings of reduced P3 amplitudes
Holcomb et al. [19]	Increased P3 latency, decreased amplitude in ADHD	Consistent with delayed P300 latency and reduced amplitude

Although significant differences in MMN and P300 components were observed between children with ADHD and healthy siblings, these neurophysiological measures did not correlate significantly with the severity of ADHD symptoms as assessed by the Connors' ADHD Rating Scale. This dissociation may reflect the complex and multifaceted nature of ADHD, where neurophysiological dysfunctions measured by ERP components capture specific cognitive processing deficits, such as attention allocation and sensory discrimination, that do not directly parallel behavioral symptom severity reported by caregivers. Variability in symptom reporting, the multidimensionality of ADHD symptoms, and potential compensatory neural mechanisms may further contribute to this lack of alignment.

Additionally, sample size limitations and the cross-sectional design may reduce sensitivity to detect such associations. Future longitudinal studies incorporating multimodal assessments and larger, heterogeneous cohorts are needed to elucidate the relationship between neurophysiological markers and clinical symptomatology more clearly, which may enhance understanding of ADHD endophenotypes. This study involved multiple comparisons across several electrode sites and ERP components. We did not apply formal corrections for multiple testing, increasing the potential for Type I errors. However, the large effect sizes observed provide support for the robustness of key findings. Nonetheless, replication in larger cohorts with multiple comparison adjustments is warranted. While the small sample size (n=28) limits generalizability, the large magnitude of group differences underscores the robustness of the observed neurophysiological abnormalities. It is important to note that the exclusion of participants with excessive movement may have led to a sample with less pronounced hyperactivity symptoms, which should be considered when interpreting the results. Future investigations are encouraged to employ larger, more inclusive samples and conduct formal power analyses to strengthen the evidence base.

## Conclusions

The present study demonstrates significant neurophysiological differences in children with ADHD, characterized by prolonged latencies and reduced amplitudes in the MMN and P300 components of ERPs across multiple electrode sites. These findings indicate alterations in cognitive and attentional processing associated with ADHD. However, rather than definitive objective markers, ERPs should be regarded as promising candidate biomarkers that require validation through replication in larger, more diverse, and clinically heterogeneous cohorts.

Notably, the absence of significant correlations between ERP parameters and ADHD symptom severity highlights an important limitation of this study. This dissociation suggests that ERP abnormalities may reflect specific neurocognitive dysfunctions or endophenotypes that are not directly aligned with the overall behavioral symptom severity assessed by clinical rating scales. Consequently, ERPs should be viewed as complementary tools to clinical assessment rather than standalone diagnostic measures. Future research should focus on standardizing ERP methodologies, verifying these findings in larger samples, and exploring their utility in improving diagnostic accuracy, treatment monitoring, and longitudinal outcome tracking in ADHD populations.
